# One Health: The global challenge of epidemic and endemic leishmaniasis

**DOI:** 10.1186/1756-3305-4-197

**Published:** 2011-10-10

**Authors:** Clarisa B Palatnik-de-Sousa, Michael J Day

**Affiliations:** 1 Laboratório de Biologia e Bioquímica de Leishmania, Instituto de Microbiologia "Paulo de Góes", CP 68040, 21941-902. Universidade Federal do Rio de Janeiro, (UFRJ), Rio de Janeiro. Brazil; 2 School of Veterinary Sciences, University of Bristol, Langford BS40 5DU, UK

## Abstract

'One Health' proposes the unification of medical and veterinary sciences with the establishment of collaborative ventures in clinical care, surveillance and control of cross-species disease, education, and research into disease pathogenesis, diagnosis, therapy and vaccination. The concept encompasses the human population, domestic animals and wildlife, and the impact that environmental changes ('environmental health') such as global warming will have on these populations. Visceral leishmaniasis is a perfect example of a small companion animal disease for which prevention and control might abolish or decrease the suffering of canine and human patients, and which aligns well with the One Health approach. In this review we discuss how surveillance for leishmaniases is undertaken globally through the control of anthroponootic visceral leishmaniasis (AVL) and zoonotic visceral leishmaniasis (ZVL). The ZVL epidemic has been managed to date by the culling of infected dogs, treatment of human cases and control of the sandfly vector by insecticidal treatment of human homes and the canine reservoir. Recently, preventive vaccination of dogs in Brazil has led to reduction in the incidence of the canine and human disease. Vaccination permits greater dog owner compliance with control measures than a culling programme. Another advance in disease control in Africa is provided by a surveillance programme that combines remote satellite sensing, ecological modelling, vector surveillance and geo-spatial mapping of the distribution of vectors and of the animal-to-animal or animal-to-human pathogen transmission. This coordinated programme generates advisory notices and alerts on emerging infectious disease outbreaks that may impede or avoid the spreading of visceral leishmaniasis to new areas of the planet as a consequence of global warming.

## Review

### What is visceral leishmaniasis?

Visceral leishmaniasis (VL) is a chronic and frequently lethal disease caused by protozoan parasites of the *L*. *donovani *complex, order Kinetoplastida. The aetiological agents are: *L. donovani *in India and Central Africa and *L. infantum *in the Americas, the Middle East, Central Asia, China and the Mediterranean. The human disease is lethal if not treated early after the onset of clinicopathological abnormalities that include: malaise, anaemia, cachexia, hypergammaglobulinaemia, hepato-splenomegaly and progressive suppression of the cellular immune response. *L. donovani *complex species are intracellular parasites of macrophages of lymphoid organs such as the spleen, lymph nodes, bone marrow and liver. Their biological cycle alternates between the amastigote form in the vertebrate host and the promastigote form in the gut of the sandfly vector. More than 90% of human cases of VL occur in India, Sudan, Bangladesh and Brazil. Each year 500,000 new human cases of VL are reported [1].

Several species of vertebrate mammals may be infected naturally with *Leishmania*. Canids are the main reservoirs for the viscerotropic species in the Mediterranean, Asia, North Africa and South America. Considering the presence or absence of animal reservoirs for *Leishmania*, two basic types of epidemiological cycles are noted: zoonotic (ZVL) or anthroponotic (AVL) [reviewed in [2]].

AVL in India and Central Africa is caused by *L. donovani *and involves a severe parasitism of the blood and skin and an anthropophilic vector, making man the reservoir of the disease. In contrast, ZVL with dogs as reservoir hosts is usually associated with *L. infantum *and is found in the Americas, the Middle East, Central Asia, China and the Mediterranean. ZVL is considered a canid zoonosis in which sandflies become infected mostly by feeding on the skin of canids and humans are the final host of the parasites. Blood-parasitism in people with ZVL was at first considered not to occur [3] but has been shown recently [4,5]. Skin parasitism has also been found in human patients with severe patent infection, indicating they might also act as a competent reservoir for the parasite and be involved in the transmission cycle [6].

### Visceral leishmaniasis and the One Health paradigm

'One Health' is a concept that is firmly rooted in the history of medicine and healthcare, but that has been rediscovered over the past decade to now become a global focus in biological science [7]. One Health proposes taking a holistic view of the previously distinct disciplines of human medicine, veterinary medicine, environmental science and wildlife conservation. One Health recognizes the intrinsic links between these areas of scientific endeavour and the impact that changes in one might have upon the others. A major focus of One Health endeavours has been in the area of infectious disease. It is recognized, for example, that environmental changes (e.g. global warming, deforestation and associated urbanization) and the effects of such changes on wildlife species (e.g. survival, geographical range) may lead to the emergence of novel infectious agents or alternative means of vector transmission of such agents. As domestic animal and human populations move closer to traditional wildlife habitats, the risk of cross-species transfer of novel infectious agents increases. It is widely recognized that the majority of emerging human infections will derive from wild or domestic animal sources [8]. In order to identify, characterize and undertake surveillance for emerging infections, and to develop integrated strategies for the control and prevention of the associated diseases, a One Health approach becomes imperative. In such situations, it is essential that interdisciplinary teams of medical, veterinary and environmental scientists work together to address the problems. The One Health concept has now been widely endorsed by key organizations such as the World Health Organization (WHO), the World Organization for Animal Health (OIE), the Food and Agriculture Organization (FAO) and the US Centers for Disease Control and Prevention (CDC). One Health is promoted through the activities of the US One Health Commission, the US One Health Initiative and the One Health Committee of the World Small Animal Veterinary Association (WSAVA) [9]. The first international One Health Conference was held recently in Melbourne, Australia and provided a global focus for this cross-disciplinary endeavour.

ZVL is a disease that epitomizes perfectly the need for a One Health approach and we hope that this will become clear in the discussion that follows. This is a disease of major human and veterinary medical significance that involves a complex interplay between a protozoal pathogen, arthropod vectors, environmental influence on vector distribution, a small companion animal (dog) reservoir of infection and susceptible human populations. The effective control of ZVL will essentially involve interdisciplinary teams of microbiologists, parasitologists, entomologists, ecologists, epidemiologists, immunologists, veterinarians, public health officers and human physicians. More importantly, the One Health approach fits perfectly the requirements for surveillance and control of this infection. The present review examines the current state-of-the-art in surveillance, control and prevention of VL, but also highlights the deficiencies in these areas that might be addressed through application of One Health principles.

### How surveillance for leishmaniasis is done globally through the control of the anthroponootic human disease (AVL)

Geographically, VL occurs in 88 countries around the world, of which 60 per cent of disease foci are in well-defined areas of Bangladesh, India and Nepal. In AVL, untreated patients are the sole sources of infection for the vector [10]. These foci of AVL are the origin of the most severe and deadly epidemics. In the 1960s, during the malaria eradication programme and due to the adoption of intensive vector control measures AVL was almost eliminated in this region, illustrating the vulnerability of the transmission cycle. The control/elimination strategy for AVL is based on aggressive identification of cases, effective management and vector control measures for reduction of, not only morbidity and mortality, but also disease transmission. The use of simple, reliable and inexpensive tests for field-level serological diagnosis, new orally-administered drugs and long-lasting insecticide-impregnated bed nets is expected to substantially decrease the number of cases, reduce transmission and prevent epidemics. In 1991-1992, an enhanced effort by the Government of India combining widespread availability and easy accessibility of drugs (antimonials), and spraying of houses with DDT, led to a 67.7 percent reduction in morbidity and 73.3 percent reduction in mortality by 1995 [10].

Recently, the governments of India, Bangladesh and Nepal launched an AVL elimination initiative, aiming to reduce the annual incidence to less than 1/10,000 population by 2015 [10]. Bihar, being the second largest populated State in India, has reported as many as 200,000 deaths from AVL since 1977. The burden of disease in India was estimated as 15 and 11 thousand new cases per year in 2001 and 2002, respectively. Approximately 1850 cases occur in Nepal annually and 30,000 in Bangladesh [10]. The difficulty of controlling AVL in the past in this region was attributed to a combination of factors including: lack of political will, non-availability of reliable diagnostic tests and unresponsiveness to antimonials, as well as inadequate vector control. The second-line drug amphotericin B is prohibitively costly and requires intravenous administration and hospitalization. In patients recovered from AVL, post-infection dermal leishmaniasis is recognized and these individuals may act as a potential reservoir of infection. The proposed control strategy exploits recent technological developments in diagnosis, drug development and vector control. The transmission pattern in this region involves *P. argentipes *as the sandfly vector [10].

Africa is also one of the main areas of AVL and has lately experienced the benefit of very modern methods of disease surveillance. The Armed Forces Health Surveillance Center, Division of Global Emerging Infections Surveillance and Response System Operations, USA (AFHSC-GEIS) [11] initiated a coordinated, multidisciplinary programme to link datasets into a predictive surveillance programme that generates advisory notices and alerts on emerging infectious disease outbreaks. The activities of this group combine: (1) satellite remote sensing and ecological niche modelling for ecological and climatic events that influence the potential for disease outbreaks; (2) arthropod-vector surveillance and geo-spatial mapping for characterizing vector presence, abundance and disease transmission capability; and (3) animal-host surveillance for detecting vector and pathogen exposure events and animal-to-animal or animal-to-human pathogen transmission [11]. This model has successfully predicted outbreaks of Rift Valley fever. The WHO and FAO are currently using the programme's Rift Valley fever advisory notices and alerts to prepare for and mitigate the impact of outbreaks of Rift Valley fever in Africa [11]. The National Aeronautics and Space Administration (NASA) Goddard Space Flight Center monitors global-scale indicators of climate variability and generates data on land surface temperature, normalized difference vegetation index, sea surface temperature and rainfall that signal the persistence of an eco-climatic trend with known associations to disease outbreaks. A risk assessment is performed on the basis of these data. While the programme's vector surveillance activities are centred historically on mosquitoes (Rift Valley fever and Malaria), in 2009 they expanded to include the sandfly vectors of leishmaniasis [11]. Large-scale surveillance of over 200 sites in Kenya captured 3,500 sandflies that were tested for *Leishmania *spp. infection using polymerase chain reaction (PCR) techniques. This traditional surveillance work forms the basis for future predictive surveillance for leishmaniasis in that country and potentially elsewhere in sub-Saharan Africa [11]. To date, there have been two major findings with epidemiological and predictive surveillance implications in Kenya. Firstly, *Leishmania major*-infected sandflies were detected in two regions not previously known for *Leishmania *transmission: Isiolo and Lamu. Secondly, *Phlebotomus orientalis*, a known vector of VL in Sudan, but rare in Kenya, was detected in large numbers in Isiolo and in Garissa [11]. Furthermore, reports from the northeast of Kenya on possible cases of AVL among refugees from Ethiopia and Somalia started to occur after the 1997, 2000-2001 and 2006-2007 El Niño rainfalls and possibly historically since the 1930s [11]. If a temporal relationship exists between El Niño events, sandfly activity and outbreaks of leishmaniasis this finding will provide a solid foundation for expanding predictive surveillance capability in Kenya beyond the Rift Valley fever project [11].

### Zoonotic visceral leishmaniasis (ZVL)

ZVL is one of the most important emerging diseases [12] that is caused by *Leishmania infantum *and transmitted by phlebotomine sand flies from a canine reservoir [6]. American and European ZVL affects mainly children and young adults [reviewed in [2]]. In Europe the majority of cases occur in adult patients with HIV infection or symptomatic acquired immunodeficiency syndrome (AIDS) [13] or in organ transplant recipients with immunosuppression [14] and in children [15].

Epidemiological studies of ZVL indicate that there appears to be an increased prevalence of *L. infantum *infection with increasing age of dogs [16-19], although this was not the case in a study of 33,937 dogs in Brazil [20]. No specific canine gender predisposition has been described for ZVL in several endemic countries [16,19,21,22]; however, in France a greater prevalence of ZVL was found in male dogs [17] and in an endemic area of Brazil high rates of seropositivity were found among male animals [23].

The German shepherd dog [18,21] together with the boxer [18] and doberman [19,21] appear to be dog breeds predisposed to infection in France, Portugal and Greece. In contrast, in Greece dogs of the collie breed are infected rarely [19] and there is a well-known resistance in Ibizian hounds in Spain [24]. In Brazil however, the most affected breeds are the long-coated cocker spaniel (26.9%) and the short-coated boxer (24.6%) [20], while in Italy no breed-related predisposition is reported [16]. A role of dog genetics in resistance or susceptibility to disease following infection appears likely. Candidate genes such as *Slc11a1 *(solute carrier family 11 member a1) [25] and the polymorphic genes of the major histocompatibility complex (MHC) [26] have been analyzed in relation to susceptibility to ZVL. *Slc11a1*, formerly known as *Nramp1*/*Ity*/*Lsh*/*Bcg*, is a proton/divalent cation antiporter that regulates susceptibility to infectious and autoimmune disease [27]. It was originally described in mice for its roles in regulating resistance and susceptibility to *Salmonella*, *Leishmania *and *Mycobacterium*. Functional studies with murine *Slc11a1 *implicate its involvement in macrophage function, including up-regulation of chemokines and cytokine genes, such as those encoding tumour necrosis factor (TNF)-α and interleukin (IL)-1β, and induction of nitric oxide synthase (iNOS) [reviewed in [28]]. Explaining the previous results of higher prevalence of infection in boxers [18,20] Sanchez Robert *et al. *[28] reported that the most frequent haplotypes of *SLc11a1 *included *TAG-8-141*, which was present in all breeds, in both case and control animals; and *TAG-9-145*, which was overrepresented in the control population and found mostly in boxer dogs. Within the boxer breed, 81% of healthy dogs were homozygous for *TAG-9-145*, whereas *TAG-8-141 *was significantly associated with case boxers. The *TAG-8-141 *genotype determined higher prevalence of the illness in the boxer breed, disclosing the importance of breed genetic background in susceptibility to ZVL [28]. A major study of the genetic basis for susceptibility to canine leishmaniasis, applying the technique of the genome-wide association study (GWAS), is nearing completion as part of the European Union-funded LUPA project [29].

Recently ZVL has become an emerging problem in some dog breeds in the USA and Canada [30,31], with an annual quantitative PCR prevalence of greater than 20% within an at-risk Foxhound population [25]. Although classically, *Leishmania *is transmitted by infected sandflies, and phlebotomine sandflies are known to exist in the USA, means of ongoing *L. infantum *transmission in North American dogs is currently unknown. Although the vertical (transplacental or transmammary) routes of transmission were not considered possible [32], several reports have indicated that endemic ZVL may be transmitted vertically [30,33]. Boggiato *et al. *[30] described disseminated *L. infantum *parasites as identified by PCR in 8-day-old pups born to a naturally-infected, seropositive American dog with no travel history. This was the first report of vertical transmission of *L. infantum *in naturally-infected dogs in North America. In Brazil, Andrade *et al. *[32] reported a kennel study with experimental infection where vertical transmission did not occur and conversely, da Silva *et al. *[33] reported the first case of vertical transmission of *L. infantum*, confirmed by PCR and immunohistochemistry techniques in two stillborn pups from a bitch naturally infected with *L. infantum*. *L. infantum *has also been transmitted between dogs venereally [34] and by blood transfusion [35].

Canine cases of leishmaniasis have also been reported in traditionally non-endemic northern European countries (e.g. The Netherlands and the United Kingdom) [36-40], where traditional sandfly vectors are thought not to exist. The large-scale movement of pet dogs under the European Pet Passport scheme, coupled with predictions on climate change in northern European countries, has led to concerns that canine leishmaniasis may become an established infection in these areas in the future [36,37].

### Impact of control of the canine reservoir on the incidence of human and canine disease

ZVL is endemic in the Mediterranean basin, Middle East and South America, and the prevalence of *Leishmania *infection can reach 67% in these areas [28]. Moreover, leishmaniasis is emerging within non-endemic areas mostly because of transportation of dogs from endemic areas and climatic changes with the expansion of the geographical range of the sandfly vector. Hence ZVL has been reported in many countries in Europe including the United Kingdom [41] and in North America particularly in English and American foxhounds and hunting dogs [31,42,43]. Cases of human ZVL have also been reported from Honduras, Venezuela, Paraguay and Argentina. Sporadic and/or imported human or canine cases have been described in Chile, Ecuador, Bolivia, Mexico, Costa Rica and French Guyana [44]. A geographically-referenced database providing links to published literature about the spatial distribution of VL was recently published by the WHO [45]

For the control of leishmaniasis the WHO recommends: (1) the treatment of human patients, (2) the culling of seropositive infected dogs and (3) the insecticidal treatment of human homes [2,12]. The epidemiological control of ZVL is performed with different tools in different countries. In the Americas, control of ZVL has proven to be particularly challenging.

Early diagnosis and treatment is essential for the human patient, but this has limited impact on transmission if the dog reservoir or insect vectors are not tackled [2,44,46]. Since dogs are the main reservoirs of the infection, a decreased incidence of ZVL in both dogs and children is found following serological screening and culling of seropositive dogs [2,47-50]. It was suggested that the incidence of human cases could be even lower if such campaigns used canine diagnostic tests with enhanced sensitivity and if there was no delay between diagnosis and culling [2,48-51]. However, as reported by Romero & Boelaert [44] and by Quinnell and Courtenay [40], the dog culling control strategy is increasingly debated. The incidence of human ZVL has remained high in Brazil despite intensive application of this strategy in recent years. Romero & Boelaert [44] also state that the elimination of ZVL in the Americas does not seem a realistic goal given the lack of political commitment, gaps in scientific knowledge, and the weakness of case management and surveillance systems [44]. Mathematical modeling suggests also that vector control and vaccination of dogs would be more efficacious than dog culling [46].

Initially, several studies correlated the practice of culling to the decrease in human and/or canine incidence of ZVL [47,49,51-54]. Magalhães *et al. *[49] defended the systematic use of dog culling, insecticide spraying and human treatment for eradication of the disease. Jerónimo *et al. *[50] reported a concomitant decrease in human pediatric cases of ZVL and of infected dogs. Braga *et al. *[51] reported that reduction of canine seroprevalence was higher when an enzyme-linked immunosorbent assay (ELISA) was used for testing and the removal of seropositive dogs was conducted more rapidly. Using an ELISA and removal of seropositive dogs, Ashford *et al. *[47] observed a significant decrease in canine seroprevalence and human incidence of disease. De Oliveira *et al.*, [53] showed that the percentage of buildings visited during the survey, the insecticidal spraying of these buildings, and the number of annual canine serological surveys were the most effective variables in reduction of the number of human cases. Costa *et al.*, [54] described that in comparison to areas receiving only household insecticide spraying, culling dogs decreased the incidence of infection by 80% [54]. However, the mathematical model described by Dye [46] condemned the epidemiological ZVL control campaign, considering it non-efficient and indeed, at low rates of canine seropositivity, no impact on the human incidence of the disease was observed [48]. The control campaign uses for the indirect immunofluorescence test (IFT), blood eluates which are saline eluates of canine blood samples collected by the survey on Whatman filter paper. However, with higher rates of canine seropositivity, corresponding to IFT or ELISA results with dog sera, the number of infectious dogs declined interrupting the transmission and the spread of epidemics [48]. Experimental results using sera instead of blood eluates lead to a 57% decrease in canine and 87.5% decrease in human cases [48]. Furthermore, Nunes *et al. *[55] described a decrease in the incidence of human ZVL that was correlated statistically to the euthanasia of dogs [55]. The effect of the insecticidal treatment of dogs was also considered important. A decrease in the prevalence of canine ZVL was observed after 65% permethrin spot-on treatment of dogs [56] and a significant reduction of anti-*Leishmania *antibody titres in dogs was observed in dogs using insecticide-impregnated collars [57].

On the other hand, several other experimental investigations did not support a correlation between dog culling and the reduction of human or canine incidence of ZVL [44,52,58-60]. The experiment of Dietze *et al. *[58] concluded that the elimination of seropositive dogs did not modify the incidence of human seroconversion or human disease, suggesting the possible involvement of other reservoirs of infection [58]. Moreira *et al. *[59] did not achieve a significant change in the incidence of canine infection despite the use of a more sensitive ELISA on serum samples; a reduced interval from serodiagnosis to removal of dogs and a higher proportion of screened dog population. The immigration of infected dogs into the study area was considered an important factor [52,59]. De Souza *et al. *[60] not find statistically significant differences on human infant seropositivy between an untreated area, an area subjected to insecticide spraying and an area subjected to the combination of insecticidal spraying and elimination of seropositive dogs [60].

Despite the fact that dogs are considered to be the main reservoir for *L. infantum*, the vector *Lutzomyia longipalpis *is opportunistic and can feed on blood from humans [6], opossums and oxen [61] making these animals also potential reservoirs. Indeed, the ability to transmit infection has been confirmed in humans, in the crab-eating fox *Cerdocyon thous*, in oppossums of *Didelphis *spp., in the black rat *Rattus rattus *and in the domestic cat *Felis catus *however, no xenodiagnosis results confirm their relevance as primary or secondary reservoirs [reviewed in [40]].

In a very detailed review of the control of ZVL, Quinnell and Courtenay [40] reported that despite the large investment in dog culling, very few randomized, controlled studies assessing its efficacy have been performed and that these intervention trials did not reduce transmission to zero [40]. Most of these studies were undertaken in Brazil. The overall proportion of infectious dogs was however considered to be higher in Europe than in South America, reflecting a higher proportion of infected sandflies in Europe and therefore suggesting a greater susceptibility of *P. perniciosus *than of *L. longipalpis *[40]. Furthermore, an alternative means of transmission is suggested to occur in Cyprus where ZVL was nearly eradicated by dog and vector destruction in 1996 and where a nine-fold increase in canine seroprevalence was observed 10 years later, with human cases mainly caused by *L. donovani *and not by *L. infantum*. This finding indicated that there were two transmission cycles running in parallel on the island: in dogs with *L. infantum *infection and in humans with *L. donovan*i infection [62].

An extreme example of the efficacy of dog culling is the case of China that had approximately 530,000 human cases of ZVL in 1951, but only 48 cases registered in 1979 after a nationwide campaign that involved mass treatment of patients, elimination and prohibition of reservoir dogs and widespread spraying of insecticides [63,64]. However, since the campaign was not systematically sustained, in 2008 a new outbreak of ZVL in children of Jiashi county was reported and investigated [63]. These campaigns are very expensive and laborious and their efficiency and feasibility are frequently reviewed and, for humane reasons lack compliance of the dog owners [12].

### How global surveillance for leishmanioses is linked to the control of the dog disease (ZVL)

In America and Europe ZVL is a canine-human zoonosis transmitted via an arthropod vector. Surveillance and control of the disease in the canine population should therefore then be a powerful tool for reduction of availability of parasites to sandflies and for the subsequent reduction in incidence of the human disease. At present, the removal of infected dogs is undertaken systematically only in Brazil [2,44] and may eventually extend to other South American countries [44]. In China dog removal is now performed only after outbreaks of ZVL in defined epidemic regions [63]. On the other hand, limited studies have been performed in the Mediterranean basin to evaluate the decrease of canine and human infection with different control methods or with treatment. Treatment of individual pet dogs is intensively practiced in Europe [65] and reduces clinical disease, parasite load and infectivity to sandflies [65,66]; however, treatment does not prevent relapse of disease [67]. It is likely that a combination of control methods will be required for eradication of the disease in the Mediterranean.

In Brazil, the methodology for epidemiological surveillance of ZVL is based on the classification of geographical areas with or without transmission of the disease [68]. The most recent approach of this program includes identification of "silent" regions where occurrence of human or canine cases of ZVL has never been reported and consequently no control measures have ever been applied [68]. There are also other areas under investigation that are considered potentially susceptible to ZVL. These include: (1) vulnerable areas with no autochthonous cases of human or canine ZVL, but that are contiguous to or located in the same road axis as municipalities with ZVL or that have a high migratory flux; (2) non-vulnerable areas, which lack the former conditions; (3) receptive areas that after entomological surveillance showed the presence of the specific insect vectors *L. longipalpis *or *L. cruzi *and (4) non-receptive areas which lack these insect vectors [68]. In areas with active transmission of ZVL, the average number of human cases over the past 5 years is used as a monitoring indicator [68]. A stratification was performed into five categories: (1) areas with the first registered autochthonous human case; (2) areas of sporadic disease (> 0.1 and < 2.4 human cases), (3) areas of moderate disease (≥ 2.4 and < 4.4 human cases), (4) areas of intense transmission of disease (≥ 4.4 human cases) and (5) outbreak areas, which are municipalities showing a number of cases greater than expected. This stratification is revised annually based on the average incidence over the last 5 years [68].

It is noteworthy that in Brazil, until 1993 95.4% of ZVL cases were restricted to the north and north east of the country, while in 2003, these areas had only 78.1% of the cases and there was an increase to 21.8% of ZVL cases in the south east and central west region of the country [68]. This expansion was a consequence of the construction of a major road that crosses the country and that allowed the migration of people with their infected dogs to new areas with the appropriate sandfly vector. Canine epidemics always precede human epidemics [69] and this is what occurs currently in the Brazilian states of Matto Grosso do Sul, São Paulo and Minas Gerais (south east and central west of Brazil) [68].

One of the preventive actions of the control campaign in Brazil is the monitoring of the sandfly vector in human residences and peri-domestic areas in order to evaluate the seasonal distribution of *L. longipalpis *and/or *L. cruzi*, so as to define the highest transmission periods and to inform prevention by chemical control of the vector. This control is achieved by spraying with pyrethroids (e.g. deltamethrin, lambdacyalothrin, alfacypermethrin, cypermethrin, cyphluthrin and betacyphluthrin) [70]. The use of deltamethrin-impregnated collars in dogs and of nets (with or without insecticides) in human homes and dog kennels is recommended, but these are not used as official tools.

A second preventive tool for ZVL in Brazil is control of the canine reservoir by removal and humane destruction of the *Leishmania*-seropositive and/or infected dogs and elimination of their carcasses [70]. To verify the presence of the canine disease a serological survey is performed. If the result is negative the areas are monitored by biannual serosurveys. If, on the other hand, the canine disease is present, the parasite is identified by a reference laboratory. In this case, the seropositive dogs are humanely destroyed and preventive actions are started by Public Health officers. If the case is autochthonous, the active search for and humane destruction of seropositive or parasite-positive dogs is performed; education of dogs owners is promoted and a serosurvey is performed in areas where the prevalence of seropositivity in the canine population is above 2% [70].

Finally, any human cases are diagnosed and treated as swiftly as possible. The identification of human cases by passive reporting or by active investigation is of paramount importance in areas of higher risk or where access of the population to treatment is problematic [70].

The low acceptance of culling of companion animals by their owners, the ethical dilemmas of veterinarians and humane reasons demand the development of alternative preventive tools. Mathematical modelling suggests that vector control and vaccination of dogs would be more efficacious than dog culling [46]. Limited data are available to judge whether treatment of individual dogs is an effective strategy for controlling infection.

Many *Leishmania *antigens have been identified as potential vaccine candidates [reviewed in [71]], but very few have been tested in field assays. First generation *Leishmania *vaccines using BCG [72] or muramyldipeptide [73] adjuvants failed to prevent ZVL in Brazil and France, respectively; while the use of alum-adjuvanted BCG resulted in a vaccine efficacy of 69.3% against the canine disease in Iran [74]. The vaccine efficacy of a double blind-control trial in the field is the result of the calculation of the incidence of disease in vaccinated subjects-the incidence of disease in controls/the incidence of disease in controls × 100. A second generation vaccine composed of *L. infantum *antigen in combination with muramyldipeptide (*Li*ESAp) had a vaccine efficacy of 92% as determined by the use of sensitive molecular diagnostic methods in an endemic area where no deaths or severe cases of ZVL occurred, indicating a low infective pressure [75]. There is only a single report of the impact of the use of a *Leishmania *vaccine on the incidence of human and canine ZVL [76]. Leishmune^®^, the first prophylactic vaccine licensed against canine visceral leishmaniasis (CVL), has been used in Brazil since 2004. The possible additive effect of Leishmune^® ^vaccination over dog culling, on the decrease of the incidence of canine and human ZVL was studied in two Brazilian endemic areas, from 2004 to 2006 [76]. In Araçatuba a decline in the incidence of ZVL in dogs of 25% was seen with a 61% decline in human cases (36 to 14 cases), indicating the additive effect of Leishmune^® ^vaccination on regular dog culling. In Belo Horizonte, where 8.1% (12,113/149,470) of the dogs were vaccinated up to 2006, the districts that had had greater vaccine coverage (85.7% of the doses) exhibited declined or sustained levels of canine and human cases of ZVL, while those with less vaccine coverage (14.3% of the doses), showed rising curves of canine and human cases of the disease. In the districts with higher vaccination levels, human cases declined by 36.5% falling from 2004 to 2006 outside of the 95% confidence interval (CI95%) of the less vaccinated districts (CI95% 2.23-21.11), which showed an average increase of 11.67%. From 1999 to 2006, the increase of canine seroprevalence and of human cases of the disease in all districts were significantly correlated (p = 0.001), confirming the importance of the dog as the infectious reservoir of the disease. The decrease in dog culling (-p = 0.007) and human incidence (-p = 0.043) were significantly correlated with the increase in the number of vaccinated animals, indicating the prophylactic impact of Leishmune^® ^vaccination on the decrease of the range of infectious dog and human populations, and so indirectly indicating a decrease in the number of dogs sacrificed. Dogs vaccinated with Leishmune^® ^did not become seroreactive in the test used by the epidemiological control campaign [76].

A vaccine called Leish-Tec^® ^has also been licensed in Brazil. It is composed of the recombinant A2-antigen of *Leishmania *amastigotes and is adjuvanted by saponin [77]. While protection due to the Leishmune^® ^vaccine has been extensively investigated in laboratory models [reviewed in [78]], explained through an immunological approach [79-82] and reported in: control versus trial-field assays with cohorts including 117 [83], 85 [84], 72 [85], and 1138 dogs [86]; immunotherapy assays with 66 [87] and 24 dogs [88]; and in 19392 vaccinated dogs in two Brazilian towns [76], there is only one report of an experimental kennel assay with Leish-Tec^® ^which was tested on seven dogs and compared with four untreated controls [77]. There is no information about the infectivity of the strain used for challenge in that study [77] and the lack of deaths in the control animals suggests that the challenge was mild. There are no reports of controlled-trial field studies with Leish-Tec^®^, but despite of the lack of peer-reviewed scientific publications, the vaccine was licensed in Brazil in 2008.

While in Brazil an average of 4,000 human cases of ZVL is reported annually, in Europe, the incidence of human ZVL is relatively lower, with a total of approximately 700 reported new cases per year for all southern European countries [89]. An estimated 2.5 million *Leishmania*-infected dogs are thought to reside in Italy, Spain, France and Portugal as determined by published serological surveys from those countries [90]. Autochthonous human and canine ZVL have spread northward, as shown by the recent reports of indigenous cases of VL in northern Italy [15] northern France [91], north western Spain [92] and southern Germany [36]. The European Centre for Disease Control is currently assessing the magnitude and importance of vector-borne diseases in Europe, mainly due to the predicted effects of global warming. Existing autochthonous vector-borne infections such as ZVL should be controlled. The risk of introducing ZVL into currently non-endemic areas of the European Union (EU) and its spread among member states was assessed for the short (2-3 years) and long term (15-20 years) [37]. In southern Europe the use of deltamethrin-impregnated dog collars [93,94] is preferred by many dog owners. Alternatively, there is substantial evidence for the efficacy of spot-on repellents containing imidacloprid, permethrin, pyriprole, metaflumizone or amitraz [95-97]. In the Mediterranean region, human and canine cases of ZVL are treated with antiparasitic drugs. In Europe, individual measures to protect dogs from sandfly bites using insecticides are commonly practiced, but no public health surveillance and control interventions such as those applied in Brazil are in place [44]. In 2011, the first European licensed canine *Leishmania *vaccine is due to be released in selected countries endemic for the disease [29]. It will be interesting to monitor the effect that this product may have on the epidemiology of the disease in those areas.

In Iran, ZVL is not controlled by serological testing and culling infected dogs, but by the use of deltamethrin-impregnated dog collars. These measures were reported to reduce the risk of infection in dogs by 54% and in children by 43% [98].

## Conclusions

The control of *Leishmania *infection in the domestic dog population is fundamental in order to avoid the spread of ZVL between dogs and man. A high level of infection in dogs, particularly in impoverished areas, is often recorded just before the start of a human epidemic and may be an important predictor of an impending outbreak of disease in the human population [69]. It is clear that identification and removal of infected dogs would reduce the prevalence of human ZVL, but recent research demonstrates that the use of insecticides in human residences and the use of insecticidal dog collars and/or the use of a preventive canine vaccine could potentially substitute for dog culling. In any case, the surveillance and control of the disease in the domestic dog is key to reducing the incidence of human disease.

The One Health concept is a worldwide strategy for expanding interdisciplinary collaborations and communication in all aspects of health care for man, animals and the environment. As monitoring of ZVL in companion animals is extremely important for the control and prevention of the human disease, ZVL is one of the best examples of a disease whose successful control and eradication depends upon the use of a One Health strategy.

## Competing interests

The authors declare that there is no conflict of interest regarding the present work and that sponsors had no role in study design, data collection and analysis, decision to publish, or preparation of the manuscript.

## Authors' contributions

CBPS did the review of the literature and wrote the article. MJD conceived the idea of the article as part of the work of the WSAVA One Health Committee and critically reviewed the article. Both authors read and approved the final manuscript.
